# Electrically Tunable Second-Harmonic Generation in High-Q Organic DAST All-Dielectric Nonlinear Metasurfaces

**DOI:** 10.3390/nano16191235

**Published:** 2026-09-30

**Authors:** Yongjie Wang, Xiaomeng Cheng, Yarong Yang, Guanhong Li, Yu Jiang, Qiang Wang, Junchen Ke, Jinping Chen

**Affiliations:** Guangxi Key Laboratory of Optoelectronic Information Processing, School of Optoelectronic Engineering, Guilin University of Electronic Technology, Guilin 541004, Chinajiangy@mails.guet.edu.cn (Y.J.);

**Keywords:** DAST organic all-dielectric nonlinear metasurface, quasi-bound states in the continuum, high Q, high SHG efficiency, electrically tunable SHG

## Abstract

Simultaneously enhancing and dynamically tuning second-harmonic generation (SHG) remains a major challenge in nanophotonics. Here, we propose a DAST-based organic all-dielectric nonlinear metasurface that combines symmetry-protected quasi-bound states in the continuum (quasi-BICs) with the strong second-order nonlinearity and electro-optic response of DAST. The quasi-BIC resonance enhances SHG efficiency by approximately 1000-fold. Applying a low voltage of 5 V significantly boosts the resonance Q-factor from 2876 to 4831. This enables an unprecedented maximum SHG conversion efficiency of 89.5% and a remarkable modulation depth of 275%. This work provides a versatile paradigm for active optical nonlinearities in organic metasurfaces, paving the way for advanced tunable nanophotonic devices.

## 1. Introduction

Nonlinear optical processes play a foundational role in modern photonics, with extensive applications encompassing on-chip light sources, quantum information processing, and high-resolution bioimaging [1,2,3]. Among these, second-harmonic generation (SHG) is a quintessential second-order nonlinear effect, in which two photons at the fundamental frequency ω are converted into a single photon at the doubled frequency 2ω through the second-order nonlinear susceptibility χ^(2)^. Driven by the trends toward device miniaturization and photonic integration, high-performance nonlinear photonics demands a transition from bulky crystals to ultracompact integrated components [4]. However, the inherently short interaction length at the nanoscale severely limits the SHG efficiency, making it difficult to achieve high-performance frequency conversion within ultracompact footprints [5]. Therefore, developing resonant structures to amplify local fields is vital for overcoming subwavelength efficiency limitations.

Metasurfaces, composed of planar arrays of subwavelength resonant elements, have emerged as a versatile platform for manipulating optical fields at the nanoscale [6]. By engineering the geometry, symmetry, and composition of meta-atoms, metasurfaces can tailor the amplitude, phase, polarization, and spatial distribution of light within an ultrathin architecture [7,8,9,10]. In nonlinear optics, plasmonic metasurfaces have been extensively investigated due to their remarkable local field enhancement at localized surface plasmon resonance (LSPR) frequencies [11]. Nevertheless, because the resonant electromagnetic fields are predominantly confined to the metal-dielectric interfaces, only the surface nonlinear susceptibility is effectively exploited. This inherent spatial restriction severely limits the conversion efficiency of nonlinear optical processes in plasmonic metasurfaces, typically to the order of 10^−11^ [12,13]. In addition, metallic metasurfaces still suffer from inherent ohmic losses and low optical damage thresholds. In contrast, all-dielectric metasurfaces, characterized by low losses and a high refractive index, effectively overcome these limitations [14]. By localizing electromagnetic fields within the bulk of the nanostructures, all-dielectric platforms allow for the full exploitation of the bulk nonlinear susceptibility, thereby significantly boosting conversion efficiencies [15,16].

Recently, bound states in the continuum (BICs) have emerged as a powerful paradigm for achieving extreme field confinement in open photonic systems. By introducing in-plane structural asymmetry, ideal non-radiative BICs can be transformed into quasi-BICs, which support leaky resonances with exceptionally high quality (Q) factors [17]. These resonances provide a giant enhancement of the local electromagnetic field, offering a compelling physical strategy to amplify SHG efficiency. Accordingly, quasi-BIC resonances have been widely adopted to enhance a variety of nonlinear optical phenomena [18,19,20,21]. To date, most demonstrations of quasi-BIC-enhanced SHG have relied on inorganic materials, such as lithium niobate (LiNbO_3_), AlGaAs, and transition-metal dichalcogenides (TMDCs) [22,23,24]. Nevertheless, in ultrathin metasurface architectures, the nonlinear conversion efficiency remains constrained by the limited interaction volume and the low second-order nonlinear coefficient. Moreover, active tunability is a crucial requirement for next-generation nonlinear photonic devices. In linear optics, researchers have integrated various external tuning mechanisms, including optical [25], thermal [26], and electrical [27,28] stimuli, to dynamically modulate resonant properties [29,30]. However, simultaneously achieving large second-order nonlinearity and strong electro-optic tunability within a single metasurface platform remains a formidable challenge. Early works demonstrated electrically tunable nonlinear optical responses in plasmonic nanocavities and metamaterials [31,32]. More recently, lithium niobate metasurfaces have achieved electro-optic SHG modulation, including Brillouin zone-folding BIC designs [33,34]. Nevertheless, achieving deep SHG modulation and high efficiency at low driving voltages remains a significant hurdle, motivating the exploration of alternative materials that combine large intrinsic nonlinearity with efficient active control. In this regard, organic nonlinear optical materials are highly attractive, as their molecular structures can provide large hyperpolarizability, strong charge-transfer interactions, and fast electro-optic responses. Among these, the organic salt DAST (4-N,N-dimethylamino-4′-N′-methyl-stilbazolium tosylate) is one of the most representative organic nonlinear crystals [35]. DAST exhibits an exceptionally large second-order nonlinear tensor component (e.g., *d*_11_ = 1010 ± 110 pm/V at 1318 nm), which significantly outperforms conventional inorganic semiconductors. Furthermore, DAST possesses a record-high electro-optic coefficient, with *r*_11_ ≈ 50 pm/V, providing a unique degree of freedom for active modulation of the optical response [36].

In this work, we leverage the synergy between the giant intrinsic nonlinearity and the large electro-optic coefficient of DAST with the extreme field confinement of quasi-BIC resonances to realize highly efficient and electrically tunable SHG in an organic all-dielectric metasurface. By breaking the in-plane symmetry of the meta-atoms, we excite high-Q quasi-BIC modes that tightly confine electromagnetic energy. As a result, the local field inside the DAST medium is greatly enhanced, which significantly increases the SHG efficiency. More importantly, by exploiting the large electro-optic coefficient of DAST, the quasi-BIC resonance can be actively tuned through an external electric field, enabling dynamic modulation of the SHG signal. We further systematically analyze the dependence of SHG enhancement on the asymmetry parameter and evaluate the electrically tunable nonlinear response. This work provides a promising strategy for realizing compact, programmable, and high-performance organic nonlinear photonic devices.

## 2. Materials and Methods

**Figure 1:**
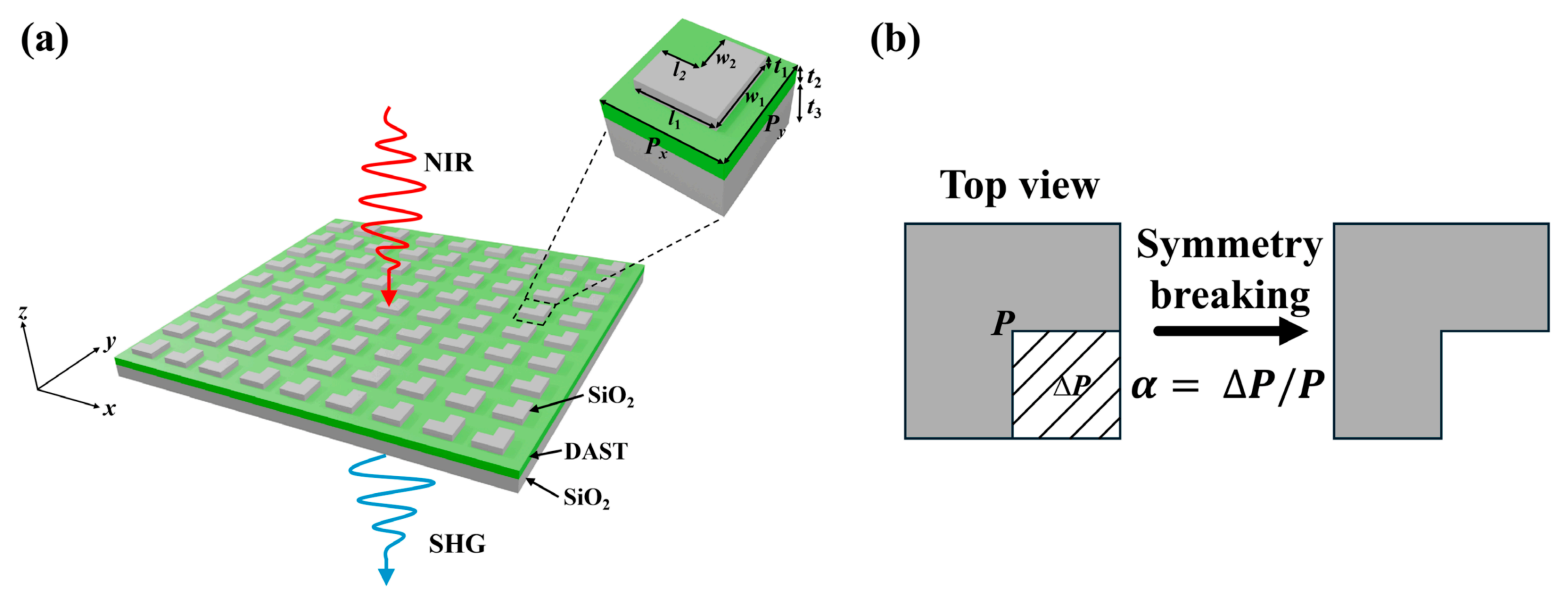
(**a**) The schematic of the SHG process in the quasi-BIC DAST all-dielectric metasurfaces. (**b**) Design of the unit cell with the definition of the asymmetry parameter α.

The DAST all-dielectric nonlinear metasurface is schematically illustrated in Figure 1a. The metasurface structure is formed by a periodic array of silicon dioxide (SiO_2_) L-shaped nanoresonators with a thickness (*t*_1_) of 200 nm and a DAST film layer with a thickness (*t*_2_) of 400 nm located on a silicon dioxide (SiO_2_) substrate (*t*_3_ = 1.2 μm). The L-shaped resonator is chosen as it simultaneously provides high-Q quasi-BICs and optimal field overlap within the DAST layer. The period of the structure is *P*_x_ = *P*_y_ = 600 nm; the side lengths of the SiO_2_ L-shaped nanoresonator are *w*_1_ = 300 nm and *l*_1_ = 300 nm, respectively. Numerical simulations of the spectral response were performed using the finite element method (FEM) via COMSOL Multiphysics (v6.0), where Floquet periodic boundary conditions were applied to the unit cell boundaries. The excitation source is an x-polarized plane wave propagating along the negative *z*-axis. The refractive index of the SiO_2_ substrate is set to 1.45. The refractive index of DAST along the a-axis was extracted from the results previously reported [37,38].

Due to its non-centrosymmetric crystal structure, DAST possesses ten non-zero components in its second-order nonlinear susceptibility tensor. The corresponding second-order polarization tensor matrix of DAST can be expressed as [39]:

(1) $$\left[\begin{array}{c} P_{x^{\prime }}^{\left(2\right)}(2\omega )\\ P_{y^{\prime }}^{\left(2\right)}(2\omega )\\ P_{z^{\prime }}^{\left(2\right)}(2\omega ) \end{array}\right]=2\varepsilon_{o}\left[\begin{array}{cccccc} d_{11} & d_{12} & d_{13} & 0 & d_{15} & 0\\ 0 & 0 & 0 & d_{24} & 0 & d_{26}\\ d_{31} & d_{32} & d_{33} & 0 & d_{35} & 0 \end{array}\right]\left[\begin{array}{c} E_{x}^{2}(\omega )\\ E_{y}^{2}(\omega )\\ 0\\ 0\\ 0\\ 2E_{x}(\omega )E_{y}(\omega ) \end{array}\right]$$

Here, ε_0_ is the vacuum permittivity. The second-order nonlinear coefficients tensor of DAST are *d*_11_ = 1230 pm/V, *d*_12_ = 166 pm/V, *d*_26_ = 135 pm/V [40]. To quantify the degree of structural symmetry breaking, we introduce the asymmetry coefficient α (0 ≤ α ≤ 1), defined as the ratio of the geometric perturbation to the original dimension (α = ΔP/P), as illustrated in Figure 1b. By systematically modulating α within the interval (0,1), the radiative leakage rate of the system can be precisely tailored. This provides a robust mechanism to manipulate the Q-factor, and, consequently, maximize the resonant enhancement of the SHG signal within the DAST-based all-dielectric metasurface.

## 3. Results and Discussion

### 3.1. Resonantly Enhanced SHG Conversion Efficiency of the DAST All-Dielectric Nonlinear Metasurfaces

**Figure 2:**
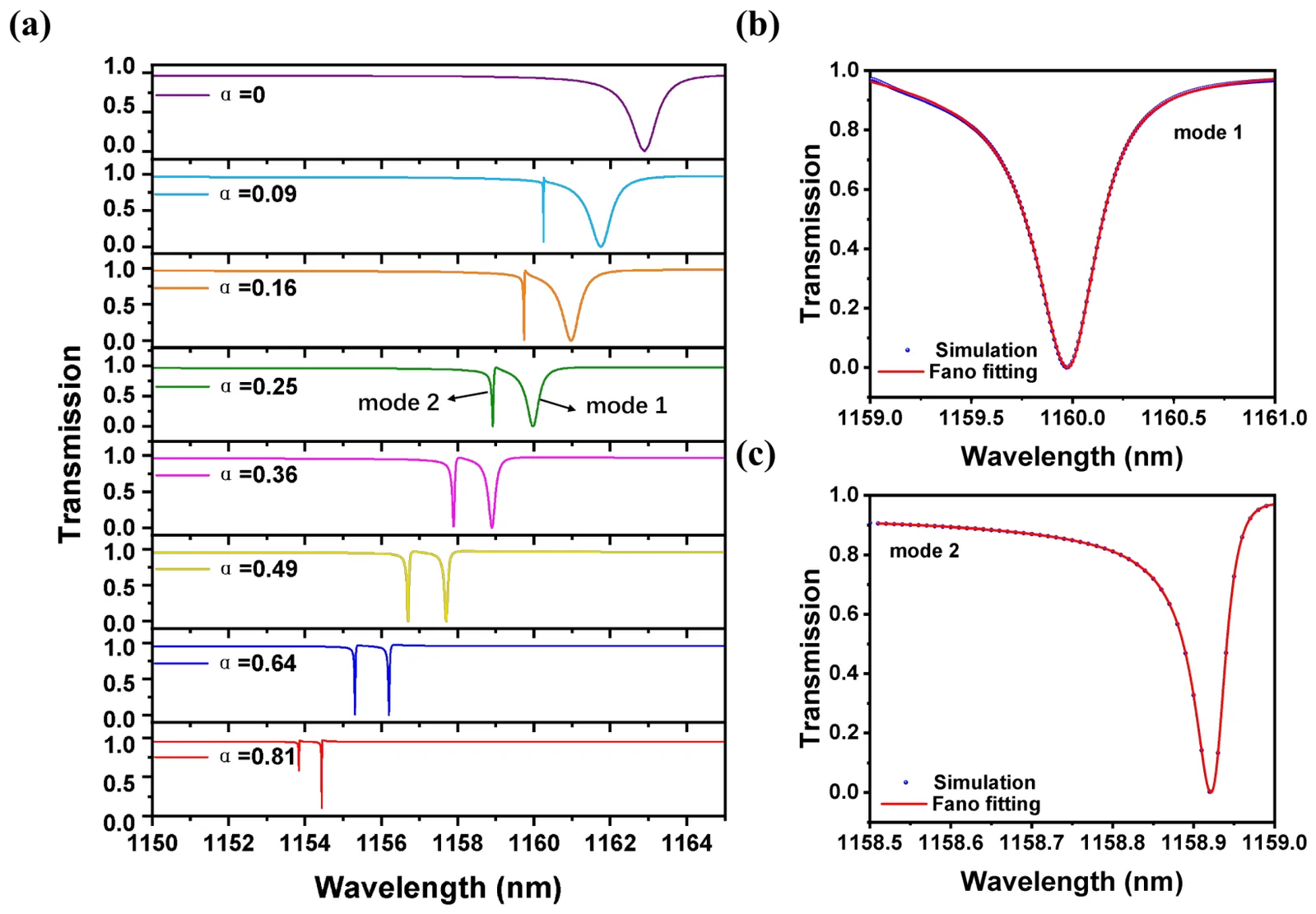
(**a**) Simulated evolution of the metasurface transmission with respect to the asymmetry α. (**b**,**c**) Fano fitting of transmission spectra of mode 1 and mode 2, respectively, at α = 0.25; the dashed line represents the simulation results, and the solid line represents the fitting results of the Fano formula.

The spectral evolution of the transmission in the DAST nonlinear metasurfaces as a function of the asymmetry parameter α is systematically illustrated in Figure 2a. For the perfectly symmetric configuration (α = 0), a single broad resonance dip is observed at approximately 1162.8 nm. As the symmetry is gradually broken (α > 0), the resonance peak of mode 1 becomes increasingly narrower as α increases. In contrast, the spectral signature of mode 2 is entirely absent at α = 0, signifying an ideal bound state in the continuum (BIC) where energy leakage to the free-space continuum is strictly forbidden, and the radiation quality (Q) factor diverges to infinity. When α ≠ 0, the structural perturbation triggers the emergence of mode 2. Specifically, at an intermediate asymmetry of α = 0.09, the transmission spectrum distinctly bifurcates into two well-defined resonance dips. This phenomenon arises because the broken in-plane symmetry establishes a radiative channel between the previously non-radiative bound state and the free-space continuum. Consequently, the BIC transforms into a quasi-BIC mode characterized by finite radiation and a high-Q factor. Furthermore, as the asymmetry α continues to increase, both of these split resonance modes exhibit a continuous blue-shift. The ability to precisely tailor the resonance positions, linewidths, and mode splitting via geometric asymmetry provides a robust mechanism for multi-band spectral modulation.

**Figure 3:**
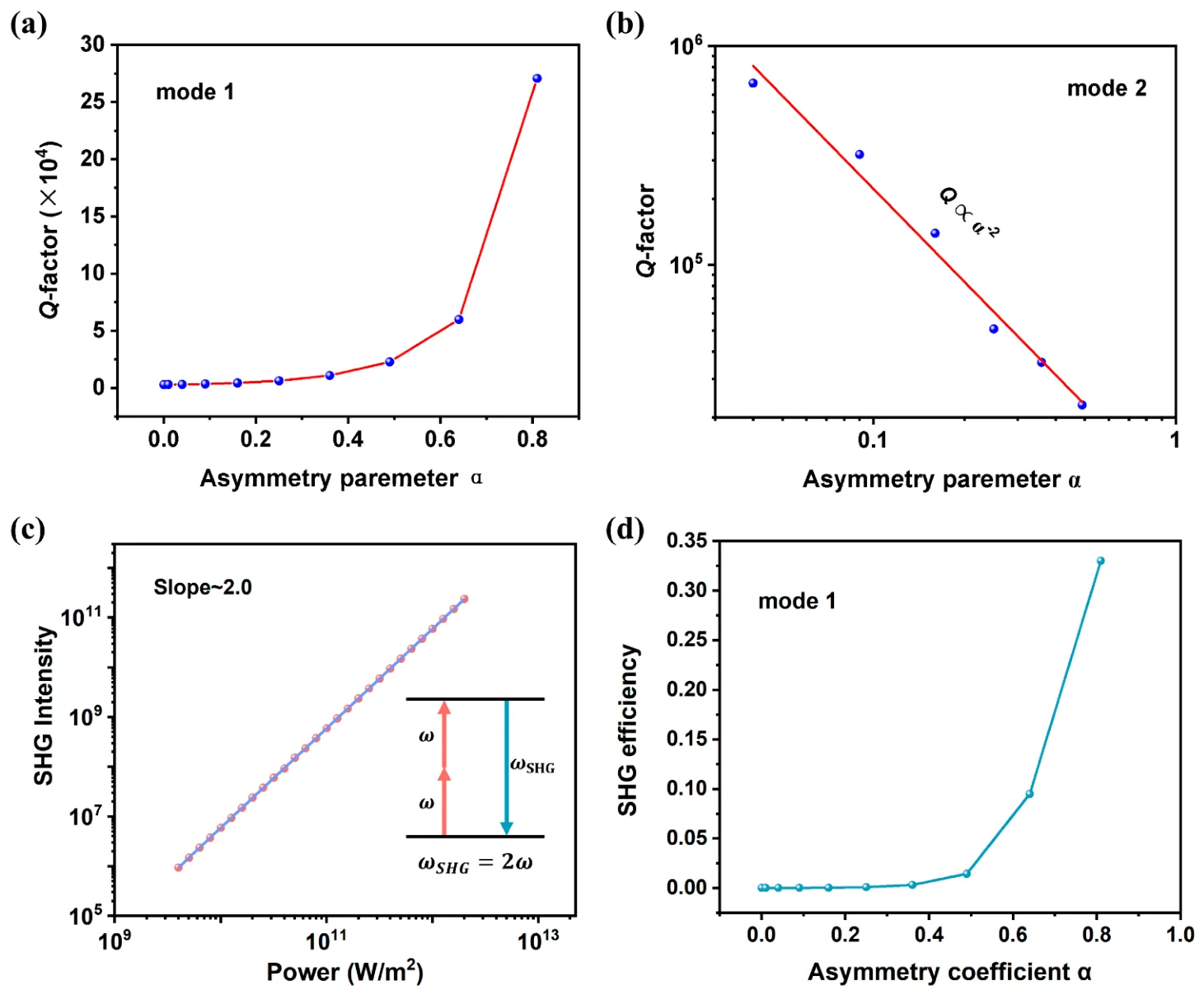
(**a**,**b**) The dependence of the Q factor of mode 1 and mode 2 on the asymmetry parameter α, respectively. (**c**) The log-log curve of SHG intensity versus excitation power. The inset shows the energy diagram of the SHG process. (**d**) The dependence of the SHG efficiency on the asymmetry parameter α of mode 1.

To elucidate how the structural asymmetry modulates the quasi-BIC characteristics, we extracted the Q-factor of the corresponding resonant modes. As shown in Figure 2b,c, the transmission spectra of modes 1 and 2 are fitted with the typical Fano formula [41]:

(2) $$T=T_{0}+A_{0}\frac{{\left[q+2(\omega -\omega_{0})/\Gamma \right]}^{2}}{1+{\left(2(\omega -\omega_{0})/\Gamma \right)}^{2}}$$

where *ω*_0_ represents the resonance peak frequency, Γ represents the leakage rate of the resonance, *T*_0_ is the background scattering parameter, *A*_0_ is the coupling coefficient for the continuous state and the discrete state, *q* is the Breit–Wigner–Fano parameter, which determines the asymmetry of the resonance spectrum. The Q-factor of the corresponding resonance is subsequently determined by the relation: Q = *ω*_0_/Γ [42]. Figure 2b,c show the results of the transmission spectrum curve fitting of mode 1 and mode 2 at α = 0.25. Mode 1 exhibits a resonance wavelength of 1160 nm with a Q-factor of 6272, while mode 2 possesses a resonance at 1159 nm with a significantly higher Q-factor of 50,824. We also investigated the connection between the asymmetry parameter α and the Q-factor of mode 1 and mode 2, as shown in Figure 3a,b. Clearly, the Q-factor of mode 1 increases with the increase in α, reaching a value of 270,646 at α = 0.81. In contrast, the Q-factor of mode 2 tends to infinity at α = 0, representing the ideal BIC state. As α increases, the in-plane symmetry of the unit cell is perturbed, allowing the BIC modes to transform into quasi-BIC modes. Notably, the Q-factor of mode 2 follows a quadratic inverse scaling law, $Q\propto \alpha^{-2}$, confirming its origin as a symmetry-protected BIC [14]. When the structural symmetry is disrupted, the bound state interacts with the continuum, resulting in the emergence of ultra-high-Q Fano resonances [41]. Notably, the resonance linewidths of mode 1 continuously sharpen while that of mode 2 broadens as α increases. This contrasting evolution of linewidths serves as a definitive hallmark of the Friedrich-Wintgen mechanism [43], indicating that mode 1 approaches an accidental BIC at larger asymmetry parameters.

The SHG conversion efficiency is defined as *η* = *P*_SH_/*P*_FF_, where *P*_SH_ is the radiative power of the transmitted SH signal collected from the substrate side, and *P*_FF_ is the incident pump power at the fundamental wavelength. To evaluate the nonlinear optical performance of the DAST all-dielectric metasurfaces, we first investigate the dependence of the SHG intensity on the incident fundamental pump power. As depicted in Figure 3c, the emitted SHG intensity is plotted against the excitation power on a logarithmic scale. The data yield an excellent fit to a linear regression with a slope of approximately 2.0. This rigorous quadratic scaling relationship unambiguously validates the second-order nonlinear nature of the observed frequency-doubling process [4]. To further explore the impact of symmetry breaking on the nonlinear optical response, the dependence of the SHG efficiency on the asymmetric parameter α is shown in Figure 3d. Notably, the SHG conversion efficiency scales positively with the degree of structural asymmetry parameter α, reaching a maximum value of 0.33 at α = 0.81. This performance is comparable to, or even surpasses, previously reported theoretical benchmarks for quasi-BIC-enhanced SHG in LiNbO_3_ metasurface (0.05) and AlGaAs (0.0286) metasurface [22,42]. The highly asymmetric geometry (α = 0.81) triggers an ultra-high Q-factor (270,646), which facilitates substantial near-field enhancement and tight spatial localization of the fundamental optical energy. Given that the SHG efficiency scales quadratically with the local electric field enhancement, the suppression of radiative leakage through symmetry breaking dramatically amplifies the nonlinear light-matter interaction at the nanoscale.

### 3.2. Resonance Performance Analysis

**Figure 4:**
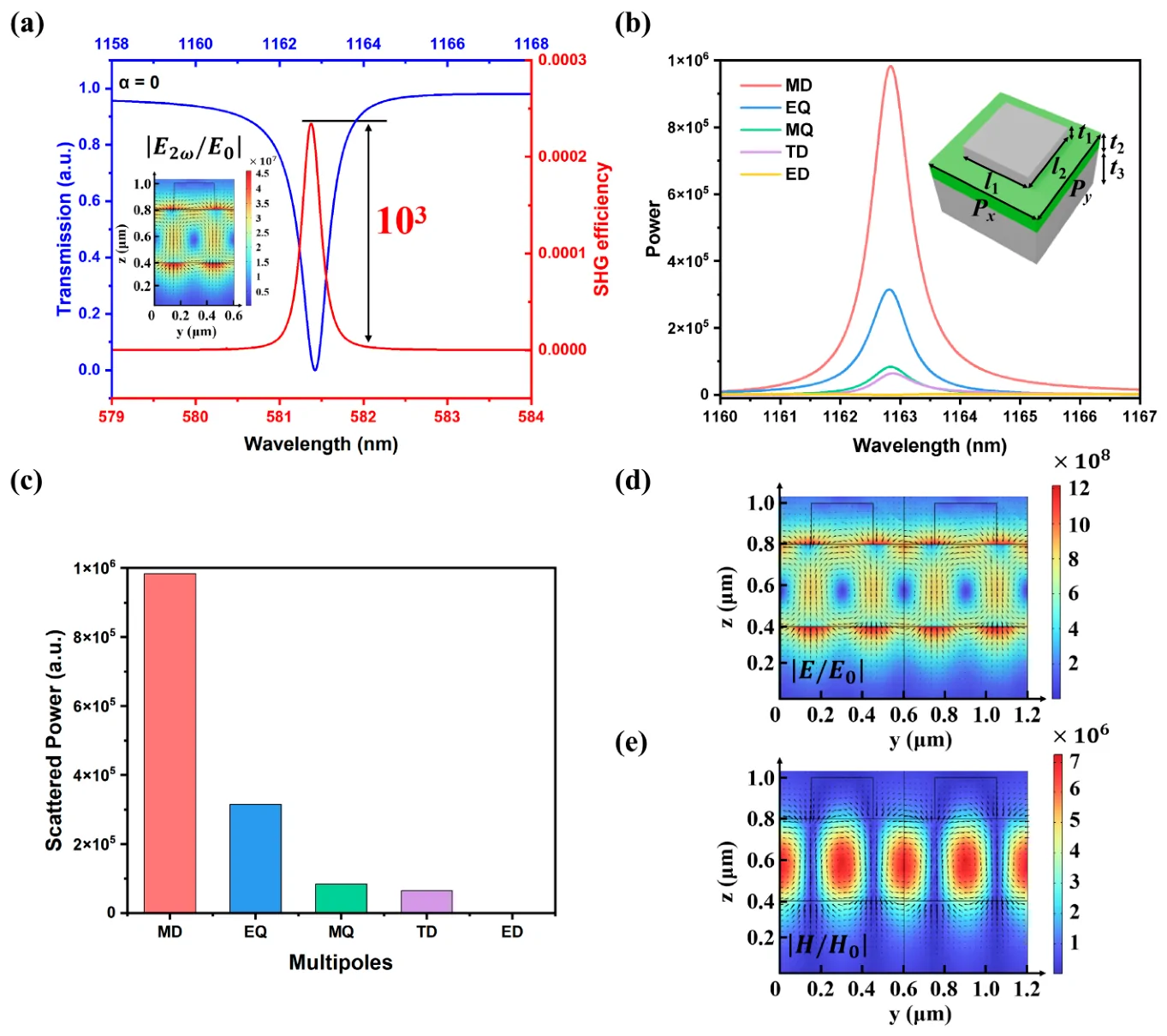
(**a**) The transmission spectrum and SHG efficiency of the DAST all-dielectric metasurface with α = 0 nm in the range of 1158–1168 nm. (**b**) Scattering powers by multipolar decomposition of the all-dielectric metasurface with α = 0 at 1162.8 nm. (**c**) Multipole scattering powers at 1162.8 nm. The electric field (**d**) and magnetic field distributions (**e**) in the y-z plane of the all-dielectric metasurface with α = 0 at the resonant wavelength of 1162.8 nm.

Figure 4a shows the transmission spectrum and corresponding SHG conversion efficiency of the DAST all-dielectric nonlinear metasurface with α = 0 in the range of 1158–1168 nm. The results reveal a striking enhancement in SHG efficiency, reaching 0.00023 at the resonance wavelength of 1162.8 nm, which surpasses the SHG efficiency at off-resonance wavelengths by three orders of magnitude. To elucidate the physical origin of this enhanced light-matter interaction, multipole decompositions of the scattering cross sections of a single meta-atom were calculated in the Cartesian coordinate system. The contributions of various multipole moments, including electric dipole (ED), magnetic dipole (MD), toroidal dipole (TD), electric quadrupole (EQ), and magnetic quadrupole (MQ), to the far-field radiation were quantitatively analyzed. As shown in Figure 4b, the resonance is predominantly governed by the MD contribution, which significantly outweighs other multipolar components. A secondary contribution from the EQ is also observed, while the ED radiation is almost entirely suppressed. Figure 4d,e show the spatial distribution of the electric and magnetic fields corresponding to α = 0. The predominance of the MD is visually confirmed by the circular displacement currents (represented by E-field vectors) localized within the DAST layer.

**Figure 5:**
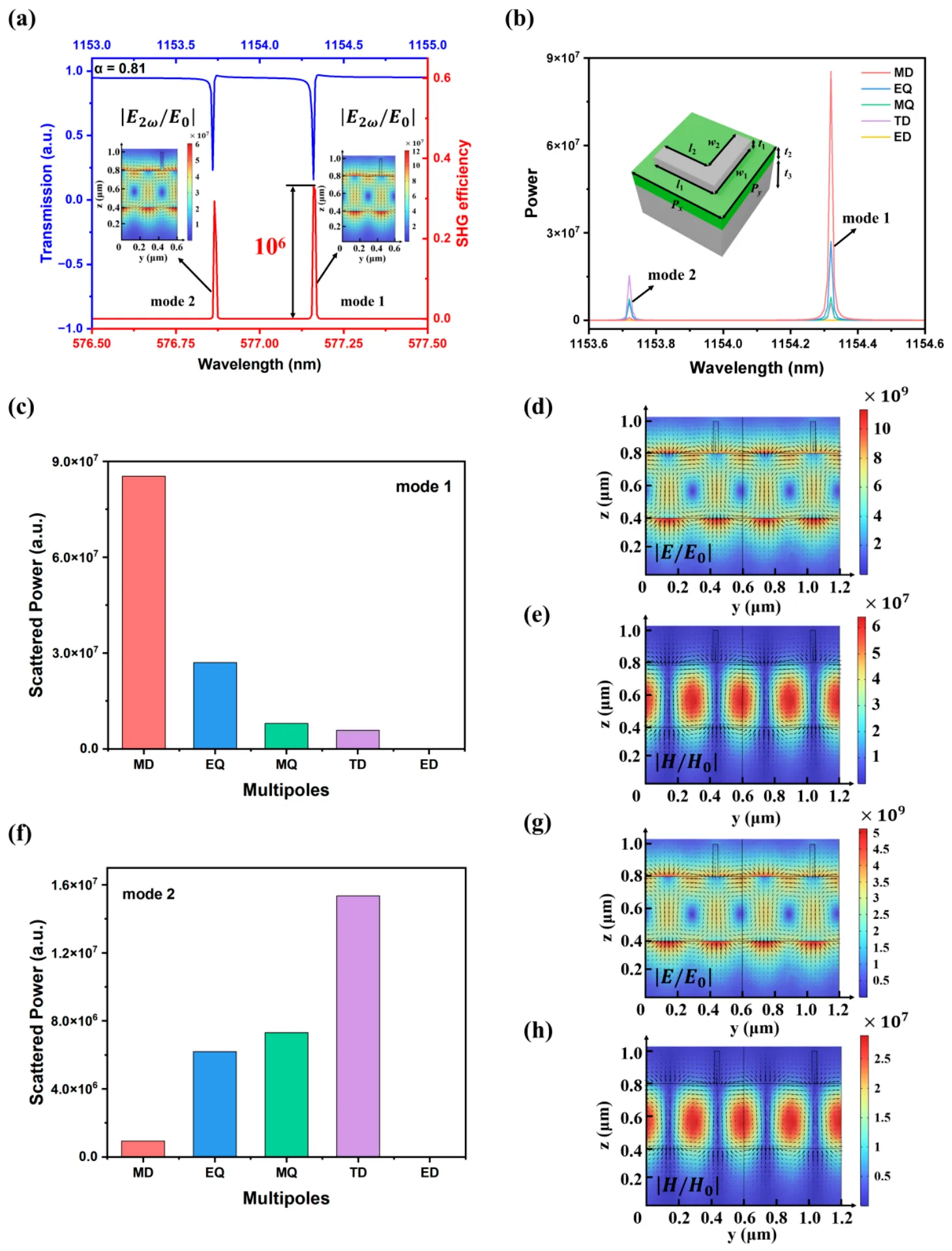
(**a**) The transmission spectrum and SHG efficiency of DAST all-dielectric metasurface with α = 0.81 nm in the range of 1153–1155 nm. (**b**) Scattering powers by multipolar decomposition of all-dielectric metasurface with α = 0.81 at the resonant wavelengths of 1153.7 nm and 1154.3 nm. (**c**) Multipole scattering powers at 1153.7 nm (mode 1). The electric field (**d**) and magnetic field distributions (**e**) in the y-z plane of all-dielectric metasurface with α = 0.81 at the resonance of mode 1. (**f**) Multipole scattering powers at 1154.3 nm (mode 2). The electric field (**g**) and magnetic field distributions (**h**) in the y-z plane of all-dielectric metasurface with α = 0.81 at mode 2.

To further illustrate the impact of symmetry breaking on the nonlinear optical response, we examine the transmission and SHG conversion efficiency of the DAST all-dielectric nonlinear metasurface at a high asymmetry parameter (α = 0.81). As delineated in Figure 5a, the transmission spectrum manifests a distinct bifurcation into two ultra-narrow resonance dips centered at approximately 1153.7 nm (mode 2) and 1154.3 nm (mode 1). This pronounced mode splitting is a direct consequence of the strong coupling between the previously hidden dark modes and the fundamental bright mode, facilitated by the high degree of geometric asymmetry. Remarkably, the SHG conversion efficiency of the DAST metasurface at α = 0.81 exhibits a three orders-of-magnitude enhancement compared to that from the metasurface at α = 0, reaching approximately 0.33 at mode 1 and 0.293 at mode 2, respectively. These values surpass the off-resonance SHG efficiency by a factor of 10^6^. This extraordinary boost in SHG efficiency is intrinsically linked to the quasi-BIC mechanism, where the suppression of radiative leakage leads to extreme near-field localization. Particularly, the electric field amplitude inside the nonlinear meta-atoms at the SH wavelengths is enhanced by a factor of 2.67. The underlying modal physics of this high-performance state is further elucidated through the multipole decomposition analysis presented in Figure 5b. For mode 1, the scattered power at 1154.3 nm remains overwhelmingly governed by the MD, complemented by a significant contribution from the EQ. Figure 5d,e present the spatial profiles of the electric field and magnetic field of mode 1 in the y-z plane, respectively. The electric field magnitude at α = 0.81 reaches an impressive order of 10^10^ V/m, representing a ten-fold increase compared to the symmetric baseline (1.2 × 10^9^ V/m at α = 0). The quintessential circulating displacement current loops with opposing rotational directions in y-z plane are characteristic of the magnetic dipole (MD) resonance [44]. The structural asymmetry significantly perturbs the field symmetry, leading to a more concentrated and intense hot-spot formation within the DAST dielectric layer. For mode 2, the resonance at 1153.7 nm exhibits a more complex multipolar composition, where the TD and MQ moments become more pronounced. Figure 5g,h show the corresponding electric and magnetic field distributions in the y-z plane of mode 2. The two electric field vortices and magnetic field with opposite rotation directions confirm the existence of TD [45]. Clearly, by introducing a high degree of structural asymmetry, both the electric and magnetic field intensities are enhanced by an order of magnitude, enabling the cavity to trap photons more efficiently and build up a much higher energy density. As claimed by Koshelev et al., the optical power at the SH wavelength P_SH_ is described by [46]:

(3) $$P_{SH}(2\omega )\propto \kappa_{2}(\omega )Q_{2}L_{2}(\omega )\kappa_{12}{\left[P(\omega )\kappa_{1}(\omega )Q_{1}L_{1}(\omega )\right]}^{2}$$

Here, *P*(ω) is the input pump power, while *Q*_1_ and *Q*_2_ denote the Q-factors of the resonances at the fundamental and SH frequencies, respectively. The terms *κ*_1_ and *κ*_2_ represent the coupling efficiencies between the far-field radiation and the bound states of the system at the pump and SH frequencies, respectively, and *κ*_12_ is the cross-coupling coefficient, which depends on the second-order nonlinear susceptibility tensor of the material. Consequently, the high SHG efficiency in the DAST metasurface at α = 0.81 is primarily governed by the high Q-factors of the fundamental and the SH resonance, the cross-coupling between the fundamental and SHG fields, and the high second-order nonlinear susceptibility of DAST. Increasing the structural asymmetry enhances the Q-factor (Figure 3a) and local fields at both the fundamental (Figure 5d) and SH frequencies (Figure 5a) of mode 1, boosting the SHG conversion efficiency to 33% (Figure 5a). Thus, symmetry breaking yields high-Q resonances, leading to a nearly 1000-fold enhancement in SHG efficiency. These results unambiguously demonstrate that tailoring the geometric asymmetry is a potent strategy for achieving extreme light-matter interactions in the nonlinear regime.

### 3.3. Influence of Structural Parameters on Transmission Performance

**Figure 6:**
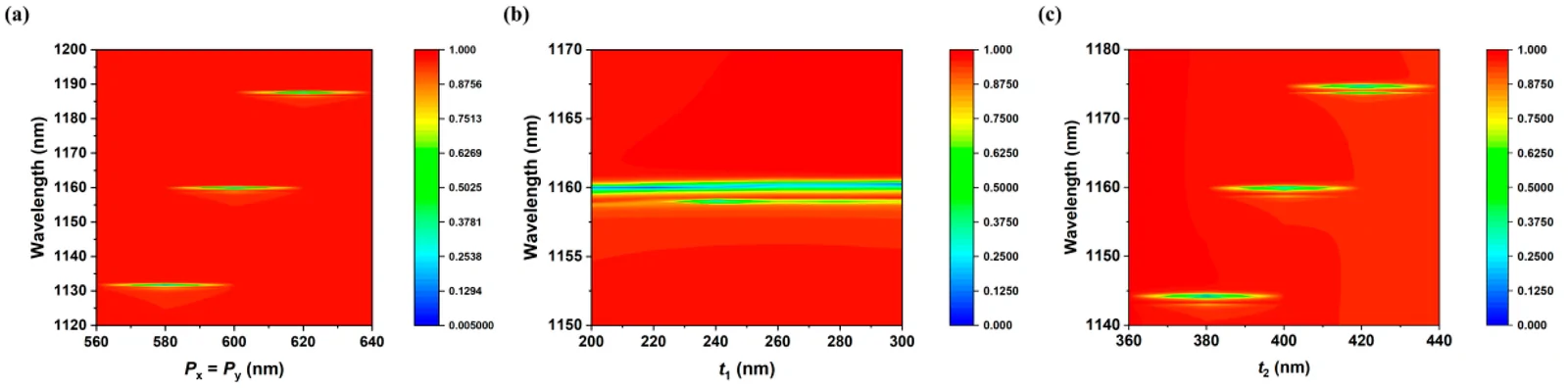
The influence of (**a**) the period P_x_ = P_y_, (**b**) t_1_, and (**c**) t_2_ on the resonance peak.

To comprehensively evaluate the spectral tunability as well as the linear and SHG responses of the proposed DAST metasurface (Figure 6), we systematically investigated the dependence of its transmission spectra on key geometric parameters. As illustrated in Figure 6a, when the lattice period (*P*_x_ = *P*_y_) is uniformly increased from 560 nm to 640 nm, the sharp resonant dip exhibits a pronounced and strictly linear red-shift from approximately 1131 nm to 1188 nm. This high sensitivity confirms that the resonance originates from a lattice-coupled guided mode, where the operating wavelength is strictly dictated by the in-plane reciprocal lattice vector. In stark contrast to this in-plane sensitivity, the transmission spectrum demonstrates exceptional stability against variations in the thickness (*t*_1_) of the top SiO_2_ L-shaped structure. As shown in Figure 6b, sweeping *t*_1_ from 200 nm to 300 nm leaves the resonance wavelength virtually unchanged at 1160 nm. The insensitivity to *t*_1_ (SiO_2_) suggests that the electromagnetic energy is deeply confined within the high-index DAST layer, minimizing the perturbative effect of the top structure. Conversely, the thickness of the middle nonlinear DAST layer (*t*_2_) plays a more significant role in modulating the resonance. Figure 6c reveals that increasing *t*_2_ from 360 nm to 440 nm induces a distinct red-shift in the resonance from 1144 nm to 1175 nm. This behavior can be attributed to the fact that the continuous DAST layer acts as the primary light-confining region for the guided resonance. Thus, expanding its vertical dimension directly increases the effective refractive index (*n*_eff_) of the supported mode, leading to a shift toward longer wavelengths. Accordingly, the resonance wavelength exhibits a higher sensitivity to the lattice period and the DAST layer thickness. As the period and the thickness of the DAST layer increase, the spatial confinement of the resonant mode is enhanced and the effective optical path is prolonged, resulting in a red-shift in the resonance position. These results suggest that the transmission and fundamental frequency response of the DAST metasurface can be precisely engineered by adjusting the asymmetry parameter α, the lattice period (*P*_x_ = *P*_y_), and the DAST layer thickness (*t*_2_), thereby enabling fine-tuned control over the fundamental electric and magnetic resonances. These findings provide a versatile degree of freedom for actively tailoring the metasurface’s response to match the desired fundamental excitation field.

### 3.4. Electrically Tunable SHG in DAST All-Dielectric Nonlinear Metasurfaces

**Figure 7:**
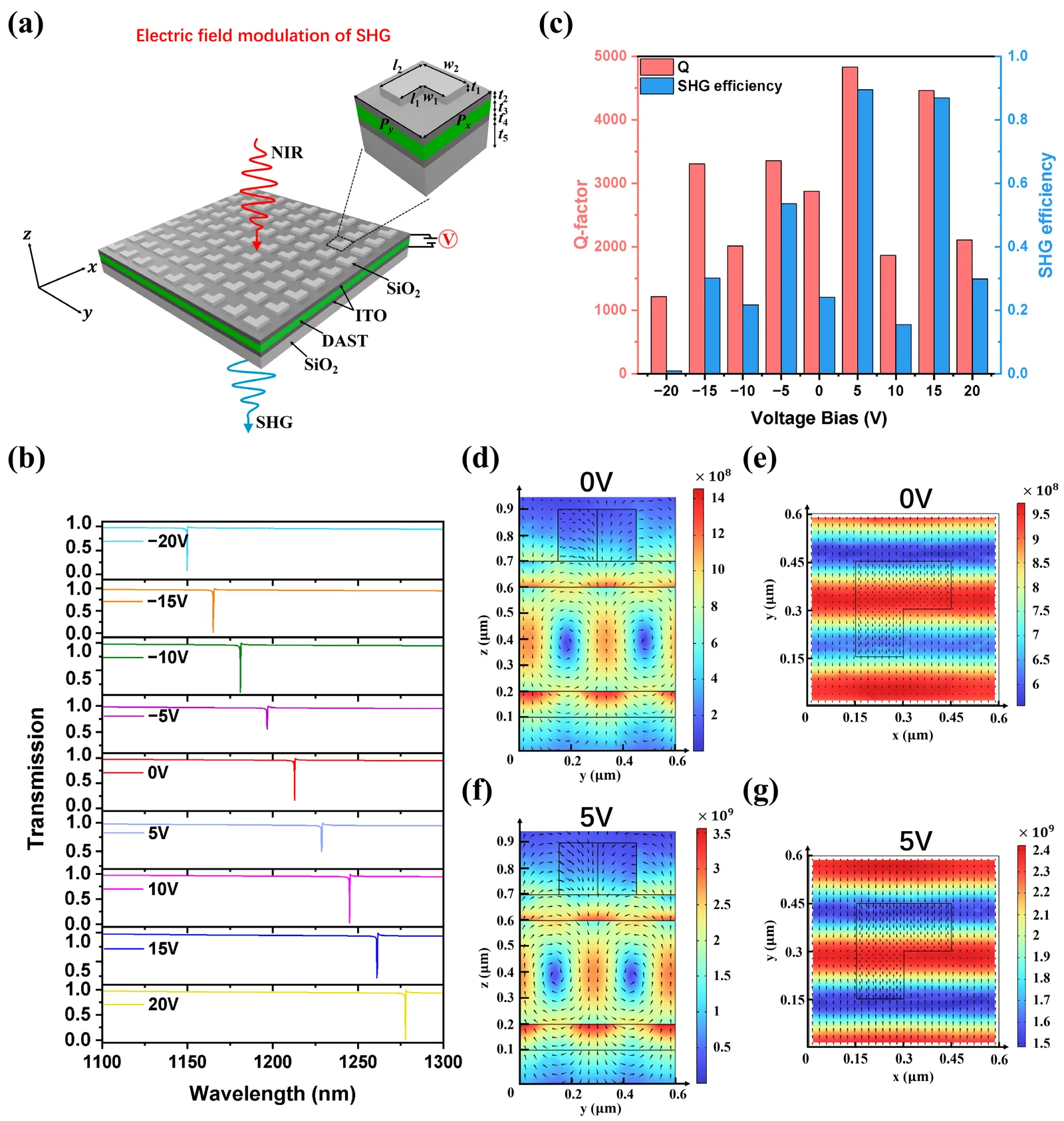
(**a**) The schematic of the electrically tunable SHG in the DAST all-dielectric metasurface. (**b**) Simulated transmission spectra under various bias voltages ranging from −20 V to +20 V. (**c**) Q factor and SHG efficiency as a function of bias voltage from −20 V to +20 V. (**d**,**e**) show the electric field distribution of the resonant wavelength at 0 V in the y-z and x-y planes, respectively. (**f**,**g**) show the electric field distribution of the resonant wavelength at 5 V in the y-z and x-y planes, respectively.

Owing to the large electro-optic coefficient of DAST, its refractive index can be significantly modulated by an external electric field [47], providing a mechanism for the dynamic tuning of the SHG response. To achieve electrical control over the SHG properties, we further designed the DAST all-dielectric nonlinear metasurface with α = 0.25 integrated with two indium tin oxide (ITO) transparent conducting films (*t*_2_ = *t*_4_ = 100 nm) as electrodes, as schematically illustrated in Figure 7a. In order to efficiently modulate the linear and nonlinear properties at a relatively low voltage, the structural parameters are set as follows: the period of the structure is *P*_x_ = *P*_y_ = 600 nm, the side lengths of the SiO_2_ L-shaped nanoresonator are *w*_1_ = 300 nm, *l*_1_ = 300 nm, *w*_2_ = 150 nm, and *l*_2_ = 150 nm, the thickness of SiO_2_ L-shaped nanoresonator (*t*_1_) is 200 nm, the thickness of DAST layer (*t*_3_) is 400 nm and the thickness of SiO_2_ substrate (*t*_5_) is 1.2 μm. Specifically, the voltage-dependent refractive index of DAST is described by [47]:

(4) $$n(U)=n_{0}+(dn/dE)\times (U/h)$$

where *U* represents the externally applied voltage, *h* is the thickness of the DAST layer, *dn*/*dE* = 3.41 nm/V, and *n*_0_ is the refractive index of the DAST without voltage. In COMSOL Multiphysics simulations, the incident light intensity is set to 1 MW/cm^2^. To simulate the infinite metasurface array, Floquet periodic boundary conditions are applied to the unit cell. The SHG output power (*P*_SH_) is determined by integrating the Poynting vector of the second-harmonic field over the transmission port at the substrate side. The SHG conversion efficiency is subsequently defined as the ratio *η* = *P*_SH_/*P*_FF_. Figure 7b displays the transmission spectra of the all-dielectric DAST metasurface under various external bias voltages ranging from −20 V to +20 V. As the bias voltage increases, the resonance wavelength (*λ*_res_) exhibits a significant red-shift from approximately 1150 nm to 1280 nm. This dynamic spectral tuning is primarily attributed to the refractive index modulation of the DAST layer via the linear electro-optic (Pockels) effect [47]. Figure 7c illustrates the calculated Q factor of the resonance (red bars) and the resulting SHG conversion efficiency ƞ (blue bars), as a function of the applied bias voltage. A strong positive correlation is observed between the Q factor and the SHG efficiency. As the applied voltage increases from 0 V to 5 V, the Q-factor of the resonance is significantly boosted from 2876 to 4831. This enhancement results from voltage-induced refractive index changes that transform the mode into a quasi-BIC with much lower radiative losses. This evolution toward a higher-Q state has a dramatic impact on the nonlinear response. Remarkably, the peak SHG conversion efficiency of 0.895 is obtained at the quasi-BIC resonance wavelength of 1228 nm under an applied bias voltage of 5 V and the SHG conversion efficiency increases from 24.2% at 0 V to 89.5% at 5 V, yielding an exceptionally high modulation ratio of $S=(\eta_{5V}-\eta_{0V})/\eta_{0V}$ = 275%. This performance significantly outperforms typical electro-optic lithium niobate metasurfaces, which have reported SHG amplitude modulations of 11.3% at ±50 V [33], SHG efficiencies of 1.2% in etchless quasi-BIC designs [48], and SHG efficiencies of 2.59% with 99% modulation ratio at 15 V [34]. These results demonstrate that DAST-based metasurfaces enable high efficient electrical control over nonlinear optical responses and low-voltage tuning. As revealed by the cross-sectional field profiles (Figure 7d–g), the transition from 0 V to 5 V shifts the metasurface into a high-Q resonance state. The electromagnetic energy becomes more tightly confined within the DAST dielectric layer, revealing a striking near-field intensity concentration in the DAST layer. In addition, the external electric field (*E*_DC_) contributes to the increase of $\chi_{eff}^{\left(2\right)}$ via the electric-field-induced second harmonic (EFISH) effect, where the effective second-order susceptibility is given by [49],

(5) $$\chi_{eff}^{\left(2\right)}=\chi_{0}^{\left(2\right)}+3\chi^{\left(3\right)}E_{DC}$$

Consequently, the dynamic enhancement of SHG efficiency under an applied bias voltage (5 V) is driven by a synergistic mechanism involving both structural resonance tuning and intrinsic material nonlinear gain. First, the applied DC voltage modulates the refractive index of the DAST layer via the electro-optic effect, which fine-tunes the electromagnetic field distribution. This drives the resonant mode toward a quasi-BIC state with lower radiative loss, boosting the Q-factor from 2876 at 0 V to 4831 at 5 V. The elevated Q-factor significantly enhances the fundamental local electric-field intensity, serving as the dominant contributor to the SHG efficiency improvement. Furthermore, the external DC field triggers the EFISH effect, which further increases the effective second-order susceptibility $\chi_{eff}^{\left(2\right)}$ of DAST. This effect provides additional gain from the perspective of material intrinsic nonlinearity. Quantitatively, the Q-factor-induced local-field enhancement contributes an approximately 2.82-fold increase in SHG efficiency, while the EFISH effect contributes an approximately 1.31-fold enhancement. Their synergistic effect yields a total ~3.7-fold enhancement of SHG efficiency. The ability to dynamically tune the SHG efficiency in DAST metasurfaces provides a crucial platform for realizing programmable and reconfigurable nonlinear optical systems.

**Figure 8:**
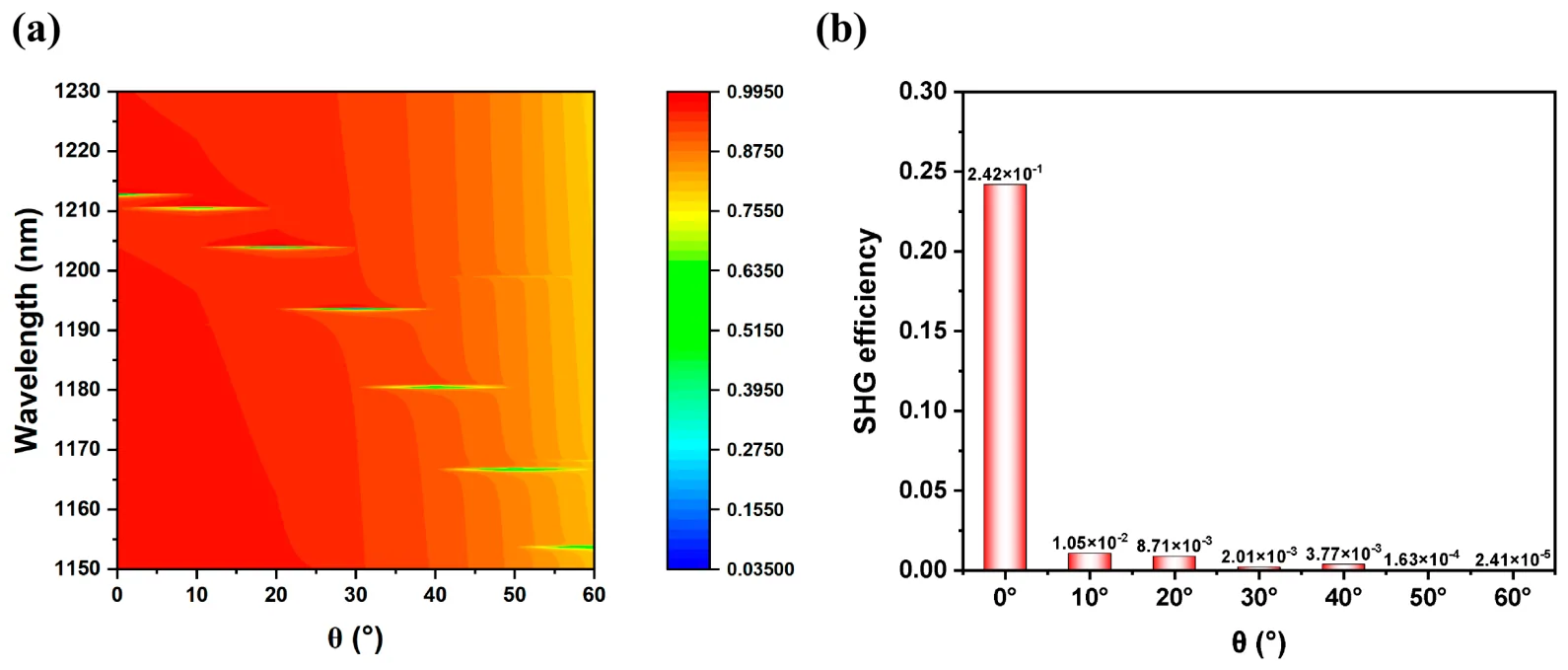
(**a**) Transmission spectra and (**b**) SHG efficiency of DAST all-dielectric metasurface at different incident angles θ.

To systematically visualize the angular dispersion characteristics of the metasurface, the transmission as a function of the incidence angle (θ) is investigated. As clearly depicted in the colormap (Figure 8a), varying the incidence angle from 0° to 60° induces a pronounced and continuous blue-shift in the resonant mode. Specifically, the transmission dip migrates from approximately 1213 nm at normal incidence (θ = 0°) down to 1154 nm at an oblique angle of 60°. This angle-dependent spectral trajectory is governed by the variation in the incident in-plane wavevector (*k*_x_ = *k*_0_sinθ) [17]. Furthermore, as the incident angle θ increases, the growing phase retardation across the structure rigorously breaks the spatial symmetry of the excitation field. This angle-induced phase gradient significantly amplifies the effective optical asymmetry of the metasurface, which in turn alters the near-field coupling and mode hybridization between electric and magnetic dipoles. Figure 8b illustrates the quantitative dependence of the SHG conversion efficiency on the incident angle θ. As the incident beam deviates from the normal axis, the SHG efficiency experiences a rapid decline. This strong angular sensitivity underscores that optimal coupling into the quasi-BIC mode and the resulting strong local field enhancement required for nonlinear generation, is strictly dictated by the incident angle.

## 4. Conclusions

In summary, we have developed an active DAST-based all-dielectric nonlinear metasurface that simultaneously enhances and dynamically tunes SHG. By breaking structural symmetry to excite quasi-BICs, we achieved extreme local field confinement at the fundamental frequencies, drastically enhancing the SHG efficiency by a factor of 1000, reaching 0.33. Moreover, the large intrinsic nonlinearity of DAST combined with its electro-optic effect enables efficient SHG modulation at low voltages from 0 V to 5 V. This electrical tuning dynamically boosts the resonance Q-factor from 2876 to 4831, driving the maximum SHG efficiency to 89.5% and delivering an exceptional modulation ratio of 275%. Our approach not only advances the fundamental capabilities of nonlinear all-dielectric metasurfaces but also lays a robust foundation for the development of next-generation active nanophotonics and integrated electro-optic devices.

## Data Availability

The original contributions presented in this study are included in the article. Further inquiries can be directed to the corresponding author.
